# [Retracted] Expression and clinical significance of hypoxia-inducible factor 1α, Snail and E-cadherin in human ovarian cancer cell lines

**DOI:** 10.3892/mmr.2025.13638

**Published:** 2025-07-28

**Authors:** Yan Zhang, Nina Fan, Jing Yang

Mol Med Rep 12: 3393–3399, 2015; DOI: 10.3892/mmr.2015.3786

Following the publication of this paper, it was drawn to the Editor's attention by a concerned reader that certain of the invasion assay data shown in Fig. 1 on p. 3395 had apparently already been submitted in different form to the journal *OncoTarget* in a paper written by different authors at different research institutes.

In view of the fact that the abovementioned data had apparently already been submitted for publication prior to their receipt at *Molecular Medicine Reports*, the Editor has decided that this paper should now be retracted from the Journal. The authors were asked for an explanation to account for these concerns, but the Editorial Office did not receive a satisfactory reply. The Editor apologizes to the readership for any inconvenience caused.

